# INTEGRATED PRIMARY CARE MODEL FOR PEOPLE LIVING WITH DEMENTIA REDUCES HIGH-RISK MEDICATIONS AND HOSPITALIZATIONS

**DOI:** 10.1093/geroni/igae098.2643

**Published:** 2024-12-31

**Authors:** Carolyn Clevenger, Anjali Khakharia, Laura Medders, Miranda Moore

**Affiliations:** Emory University, Atlanta, Georgia, United States; Emory University, Atlanta, Georgia, United States; Emory University, Atlanta, Georgia, United States; Emory University, Atlanta, Georgia, United States

## Abstract

Harmful care including the prescribing of high-risk and potentially inappropriate medications for older people is widespread among older adults, including people living with dementia (PLWD). Integrated Memory Care (IMC) is a comprehensive dementia care model where patients and their family caregivers access dementia-sensitive geriatric primary care. The IMC conducted a retrospective observational study of patients of IMC, Cognitive Neurology (CN), and Primary Care (PC) clinics aged 65 and older with a diagnosis of dementia in 2019-2021. We matched patients by age, gender and race and measured the hospitalization, rate of deprescribing and inappropriate screening test referrals using logistic regressions controlling for clinic. Additionally, we conducted a regression adjusted for state of illness (proxied by activities of daily living (ADL)) for the IMC and CN clinics. IMC patients had higher odds of deprescribing high dose antipsychotics, benzodiazepines, and opiates when compared to CN patients. IMC also had higher odds of deprescribing high dose antipsychotics, benzodiazepines, and opiates when compared to PC patients. For hospitalizations, CN had 10% [2 times] higher odds, when compared to IMC patients in unadjusted models. After adjusting for ADL scores, CN patients had 66% higher odds of having a hospital admission during the study period. Dementia-sensitive primary care reduces the rate of total and ambulatory-sensitive hospitalizations reducing healthcare costs and causing less disruption to care as well as reduces inappropriately prescribed medications among PLWD.

